# Postoperative complications predict poor outcomes only in patients with a low modified clinical score after resection of colorectal liver metastases: a retrospective cohort study

**DOI:** 10.1007/s13304-022-01312-7

**Published:** 2022-07-20

**Authors:** Hong-Wei Wang, Ke-Min Jin, Juan Li, Kun Wang, Bao-Cai Xing

**Affiliations:** Hepatopancreatobiliary Surgery Department I, Key Laboratory of Carcinogenesis and Translational Research, Ministry of Education, Peking University School of Oncology, Beijing Cancer Hospital and Institute, Haidian District, Beijing, China

**Keywords:** Colorectal liver metastasis, Complications, Modified clinical score, Hepatectomy, Outcomes

## Abstract

The aim was to identify the optimal criteria of postoperative complications (POCs) for predicting oncological outcomes after hepatectomy for colorectal liver metastases (CRLMs) and to investigate the variable prognostic implications of POCs according to the modified clinical score (M-CS). We identified 751 patients who underwent curative hepatic resection for CRLM between 2007 and 2018. Patients were categorized based on the M-CS. The impact of the severity [≥ Clavien–Dindo grade (C–D) III or comprehensive complication index (CCI) ≥ 26.2] or type [any infectious complications of POC (Inf-poc)] of POC on overall survival (OS) and recurrence-free survival (RFS) was assessed by univariate and multivariable analyses in different groups. Patients with a major or infectious complication were not associated with either RFS or OS in multivariable analysis of the whole cohort. However, patients with a high CCI had a worse OS (HR 1.51, *P* = 0.004). Among patients with low M-CS, patients with high CCI had worse OS (HR 1.49, *P* = 0.035) and RFS (HR 1.32, *P* = 0.048) than those without high CCI. In contrast, the survival disadvantage of a high CCI was not present in patients with a high M-CS. Compared to Inf-poc or major complications, a high CCI decreased long-term OS in patients treated with hepatectomy for CRLM. High CCI has a variable prognostic impact after hepatic resection for CRLM depending on the M-CS. POC is not a decisive factor to justify the use of hepatectomy for CRLM in patients with high M-CS.

**Supplementary Information:**

The online version contains supplementary material available at 10.1007/s13304-022-01312-7.

## Introduction

Hepatectomy is considered the standard of care for patients with resectable colorectal liver metastases (CRLMs). However, only a minority of patients with CRLMs are amenable to curative liver resections at diagnosis [1, 2]. Surgical indications have widened, and the introduction of local ablative procedures and effective downsizing treatments, such as selective internal radiotherapy plus chemotherapy [3–5], in recent decades have increased the conversion rate of unresectable patients with more advanced diseases and aggressive tumor biology. However, with expanded indications and more aggressive conversion therapy, severe postoperative complications may also increase [6].

A number of previous studies have shown that postoperative complications can reduce patients’ long-term survival and increase the risk of disease recurrence in patients with CRLM [7–11]. However, the postoperative complication criteria to determine survival differ among studies. Mavros et al. [7] stated that all postoperative complications were associated with shorter recurrence‐free survival (RFS) and overall survival (OS), whereas Vaz da Silva et al. [8] and Fukami et al. [9] proposed that Clavien–Dindo grade ≥ III complications predicted shorter OS. In addition, Moreno et al. [10] suggested that only infective complications affect the long-term survival of patients with CRLM. On the other hand, the comprehensive complication index (CCI) was reported to adversely impact RFS and cancer-specific survival by Yamashita et al. [11]. Moreover, most reports have drawn conclusions from nonstratified patients without regarding tumor biology or tumor burden.

Fong et al. created a clinical risk score (CRS) in 1999, and it has been frequently cited worldwide [12]. Brudvik et al. derived a modified clinical score (M-CS) on the basis of CRS in 2017. This score directly measures tumor biology and indirectly accounts for tumor burden, and the new scoring system has been demonstrated to be a better discriminator of RFS and OS than CRS in patients with CRLMs [13]. The objective of this study was to identify the optimal criteria of postoperative complications for predicting oncological outcomes and to investigate whether the impact of postoperative complications on OS and RFS is consistent in patients with different M-CS. We also wanted to evaluate whether hepatectomy should be recommended for patients with poor prognosis and high risk of postoperative complications.

## Methods

### Study population

Patients who were assigned to the first curative liver resection for CRLM between January 2007 and December 2018 at the Hepato-Pancreato-Biliary Surgery Department I, Peking University Cancer Hospital were enrolled in this study. Patients who underwent macroscopically incomplete resection of liver metastases or experienced 90-day mortality were excluded. Patients who did not receive radical treatment for the extrahepatic metastases were also excluded. Furthermore, patients who had incomplete data were lost to follow-up or received 2-stage hepatectomy were also excluded. Data were censored on 30 November 2019. The study was approved by the institutional review board.

### Patient management

In addition to routine laboratory evaluations and physical examinations, all patients underwent imaging studies using a combination of enhanced CT or MRI scans to assess the extrahepatic disease and the resectability of CRLM. A total of 12 cycles of perioperative fluorouracil-based chemotherapy was recommended unless single, metachronous resectable metastases were present or the patient refused. CRLM or primary tumor samples have been routinely analyzed for RAS and BRAF mutations since 2014. For patients undergoing CRLM resection before 2014, molecular analysis was performed on archived specimens. The details of the genomic techniques used have been described previously [14].

All surgeries were performed in a territorial center by several surgeons, each performing over 50 cancer resections per year. A standard liver resection approach was used by all surgeons. First, an exploratory laparotomy or laparoscopy was performed. Then, an intraoperative ultrasound scan was performed to locate the lesions, outline hepatic veins and portal pedicles and to look for additional tumors. A Peng multifunction operative dissector and harmonic scalpel (Ethicon Endo-Surgery, Cincinnati, OH) were used for parenchymal transection. An intermittent Pringle maneuver may be applied as determined by the surgeon. Patients complied with standard departmental postoperative care. Postoperative complications was defined as any deviation from the normal postoperative course within 30 days after hepatectomy. Postoperative complications was categorized as non‐infective or infective complications, and the severity of complications was graded using the CCI and Clavien–Dindo classification. Consistent with other analyses in the literature [7, 10, 11], Clavien–Dindo grade ≤ II and CCI < 26.2 were grouped together. Infective complications included surgical‐site infection, urinary tract infection, respiratory infection, catheter‐related bloodstream infection, and systemic sepsis.

All patients were followed up every 3 months for the first 2 years and then every 6 months thereafter. At each follow-up, CEA measurements, liver function tests, physical examinations, abdominal enhanced computed tomography or MRI scans, and computed tomography scans of the thoracic and pelvic regions were performed.

### Data collection

The following data on demographic, tumor‐specific characteristics, and surgery-related variables were recorded: sex, age, BMI, and comorbidities were evaluated by the Charlson Index scores [15] before operation, American Society of Anesthesiologists status classification (ASA), primary tumor characteristics [location (primary tumors located from the cecum to the end of the transverse colon were defined as right sided, whereas those located from the splenic flexure to the rectum were defined as left sided), depth of invasion, and lymph-node metastases], prehepatectomy chemotherapy (regime and cycles), CRLM characteristics [preoperative CEA level, synchronous vs. metachronous (synchronous CRLM was defined as liver metastasis detected at or before diagnosis of the primary tumor), tumor size, number of tumors, unilobar or bilobar metastasis, and RAS status], operative variables [blood loss, intraoperative blood transfusion, duration of surgery, extent of hepatectomy (minor or major resection), use of radiofrequency, and resection margin status (margins defined as R1 if involved microscopically or within 1 mm)], existence of extrahepatic disease, postoperative complications characteristic [infective (yes/no), complication severity (CCI and Clavien–Dindo grade)], and adjuvant treatments. Major hepatectomy was defined as resection of three or more liver segments according to the Couinaud classification, whereas minor hepatectomy comprised partial hepatectomy of less than three segments. M-CS scores were calculated in accordance with the original study (Supplementary Table 1). Two risk groups were identified as low risk (M-CS 0–1) and high risk (M-CS 2–3).

### Statistical analysis

Continuous variables with a normal distribution are described as the mean (s.d.) and compared using Student’s *t* test. Variables with a non‐normal distribution were described as the median (i.q.r.) and compared using the Mann–Whitney *U* test. Categorical variables were expressed as numbers with percentages and analyzed using the Chi-square test or Fisher’s exact test where appropriate. OS and RFS were calculated using the Kaplan–Meier method and compared using the log-rank test. The hazard ratios (HRs) of variables for OS and RFS were analyzed using the Cox regression model. Variables with *P* < 0.10 on univariable analysis were included in the multivariable Cox regression analysis. *P* < 0.05 was considered significant for all calculations. Statistical analyses were performed using SPSS^®^ version 23.0 (IBM, Armonk, New York, USA).

## Results

### Clinicopathological characteristics

During the study period, 841 patients underwent 945 liver resections for CRLM. Among them, 90 patients (10.7%) were excluded (19 patients with missing data, 30 patients with two-stage hepatectomy or only underwent repeat hepatectomy, 27 patients with incomplete resection, 10 patients lost to follow-up, 2 patients who died within 90 days, and 2 patients who died of noncancer-related reasons). A total of 751 eligible patients were divided into the two M-CS categories as follows: low M-CS group, *n* = 488 (65.0%); high M-CS group, *n* = 263 (35.0%). The demographics and tumor features of the whole cohort and the two M-CS groups are summarized in Table 1. Significant differences in patient primary tumor location (*P* = 0.003), primary tumor nodal metastasis (*P* = 0.000), CEA (*P* = 0.000), maximum tumor size (*P* = 0.000), and RAS status (*P* = 0.000) were noted between the two M-CS groups. Other oncological factors, including the number of tumors, primary tumor T stage, extrahepatic metastasis, and surgical factors, including surgical procedure and blood loss, did not differ significantly between the two groups.

**Table 1:** Patient characteristics according to different M-CS

Variables	Total (751)	Low (*n* = 488)	High (*n* = 263)	*P*
Patient characteristics				
Age (i.q.r), years	58 (51.0–64.0)	58.0 (51.0–64.0)	58 (50.0–63.0)	0.411
Sex				
Male (%)	485 (64.6)	322 (66.0)	163 (62.0)	0.299
Charlson Index scores				
Median ( i.q.r)	8(7–8)	8 (7–8)	7(7–8)	0.245
ASA, I/II/III	404/271/76	250/191/47	154/80/29	0.060
Primary tumor characteristics				
Right-sided tumor (%)	139 (18.5)	75 (15.4)	64 (24.3)	0.003
T stage				
T3 or T4 stage (%)	686 (91.3)	440 (90.2)	246 (93.5)	0.135
Node-positive primary tumor	531 (70.7)	283 (58.0)	248 (94.3)	0.000
Preoperative factors				
Preoperative chemotherapy (%)	530 (65.8)	340 (64.2)	190 (71.4)	0.502
Preoperative CEA > 20 (%)	223 (29.7)	117 (24.0)	106 (40.3)	0.000
Synchronous CLM	397 (52.9)	258 (52.9)	139 (52.9)	1.000
CRLM characteristics				
Tumor number (multiple)	477 (63.5)	316 (64.8)	161 (61.2)	0.341
Maximum tumor size ≥ 5 cm (%)	96 (12.8)	16 (3.28)	80 (30.4)	0.000
Bilateral disease (%)	367 (48.9)	248 (50.8)	119 (45.2)	0.147
Ras mutation (%)	263 (35.0)	65 (13.3)	198 (75.3)	0.000
Extrahepatic metastasis (%)	121 (16.1)	75 (15.4)	46 (17.5)	0.467
Hepatic resection				
Operative time, median (range),min	186 (140–246)	182 (136–240)	194 (145–252)	0.154
Plus ablation (%)	109 (14.5)	75 (15.4)	34 (12.9)	0.387
Major hepatectomy (%)	118 (15.7)	68 (13.9)	50 (19.0)	0.074
Blood loss (i.q.r),ml	200 (100–300)	150 (100–250)	200 (100–300)	0.083
Intraoperative transfusion (%)	61 (8.12)	37(7.58)	24 (9.12)	0.485
R1 resection	198 (26.3)	129 (26.4)	69 (26.2)	1.000
High CCI	143 (19.0)	86 (17.6)	57 (21.7)	0.106
Infectious complication	127 (16.9)	75 (15.4)	52 (19.8)	0.077
Adjuvant chemotherapy	545 (72.6)	356 (72.9)	189 (71.9)	0.797

### Postoperative outcomes

The 30‐day postoperative morbidity rate was 28.8% (216 patients). Among all patients, 127 (16.9%) developed a postoperative infective complication, 143 (19.0%) had a high CCI, and Clavien–Dindo grade ≥ III complications were observed in 87 (11.6%) patients. The details on postoperative complications are provided in Table 2. Baseline characteristics and operative variables that differed among patients with and without postoperative infective complications or any ≥ Clavien–Dindo grade III complications or had high and low CCIs are provided in Supplementary Table 2. Multivariable logistic regression analysis identified several independent risk factors associated with a high CCI, including right-sided (RS) tumors (HR 2.79, 95% confidence interval (CI) 1.75 to 4.45; *P* = 0.000), synchronous metastasis (HR 1.71, 1.10 to 2.65; *P* = 0.018), intraoperative blood transfusion (HR 8.25, 4.46 to 15.29; *P* = 0.000), operative time exceeding 180 min (HR 1.87, 1.18 to 2.09; *P* = 0.008), and major hepatectomy (HR 3.76, 2.33 to 6.10; *P* = 0.000).

**Table 2:** Postoperative morbidity

	Total (751)	CCI < 26.2(608)	CCI ≥ 26.2 (143)
Type of postoperative morbidity			
Respiratory infection	7 (0.93)	2 (0.33)	5 (3.50)
Pleural effusion	26 (3.46)	0 (0)	26 (18.2)
Pulmonary artery embolism	1 (0.13)	0 (0)	1 (0.70)
Respiratory insufficiency	2 (0.27)	0 (0)	2 (1.40)
Arrhythmia	11 (1.46)	4 (0.66)	7 (4.90)
Unstable blood pressure	10 (1.33)	9 (1.48)	1(0.70)
Cardiovascular accident	5 (0.67)	3 (0.49)	2 (1.40)
Acute liver failure	24 (3.20)	0 (0)	24 (16.8)
Biliary leak	43 (3.32)	25 (4.11)	18 (12.6)
Ascites	28 (3.73)	4 (0.65)	24 (16.8)
Upper gastrointestinal bleeding	9 (1.20)	3 (0.49)	6 (4.20)
Anastomotic leak	10 (1.33)	1(0.16)	9 (6.29)
Abdominal hemorrhage	24 (3.20)	8 (1.32)	16 (11.2)
Intestinal obstruction	12 (1.60)	4 (0.66)	8 (5.60)
Urinary tract infection	6 (0.8)	1 (0.16)	5 (3.50)
Renal dysfunction	1 (0.13)	0 (0)	1 (0.70)
Surgical-site infection			
Organ/space	67(8.92)	12 (1.97)	55 (38.5)
Incisional	10 (1.33)	2 (0.32)	8 (5.60)
Systemic infection	27 (3.60)	11 (1.80)	16 (11.2)

### Risk factors for poor outcomes in whole cohort

The median follow-up time of all patients was 30 months, ranging from 3 to 154 months. The 5-year OS rates after hepatectomy for CRLM in the whole cohort with high CCI versus low CCI, infective versus non-infective complications, and Clavien–Dindo grade ≥ III versus grade ≤ II were 32.6% versus 51.1%, 33.3% versus 49.6%, and 42.5% versus 47.5%, respectively (Supplementary Fig. 1 A–C). The 5-year RFS rates in patients with high CCI versus low CCI, infective versus non-infective complications, and Clavien–Dindo grade ≥ III versus grade ≤ II were 9.1% versus 24.8%, 12.7% versus 23.3%, and 17.0% versus 21.9%, respectively (Supplementary Fig. 2 A–C). The OS and RFS rates were significantly lower in the high CCI and infective groups compared with the low CCI and non-infective complications groups (*P* = 0.000, *P* = 0.003, and *P* = 0.000, *P* = 0.003, respectively). The OS and RFS rates in the Clavien–Dindo grade ≥ III groups did not differ significantly from those in the Clavien–Dindo grade ≤ II group (*P* = 0.078 and *P* = 0.313, respectively).

Using multivariate analysis, we identified a high CCI as a prognostic factor associated with poor OS (HR 1.51, 1.14–2.00, *P* = 0.004). Preoperative CEA > 20, primary tumor nodal metastases, RAS mutation, extrahepatic metastasis, R1 resection, and postoperative chemotherapy were also associated with OS. No significant association was found between a high CCI and RFS (HR 1.16, 95% CI 0.94–1.44, *P* = 0.166). Of note, infective complication was not associated with OS or RFS (Supplementary Tables 3, 4).

### Prognostic analyses for OS and RFS in different M-CS groups

**Fig. 1:**
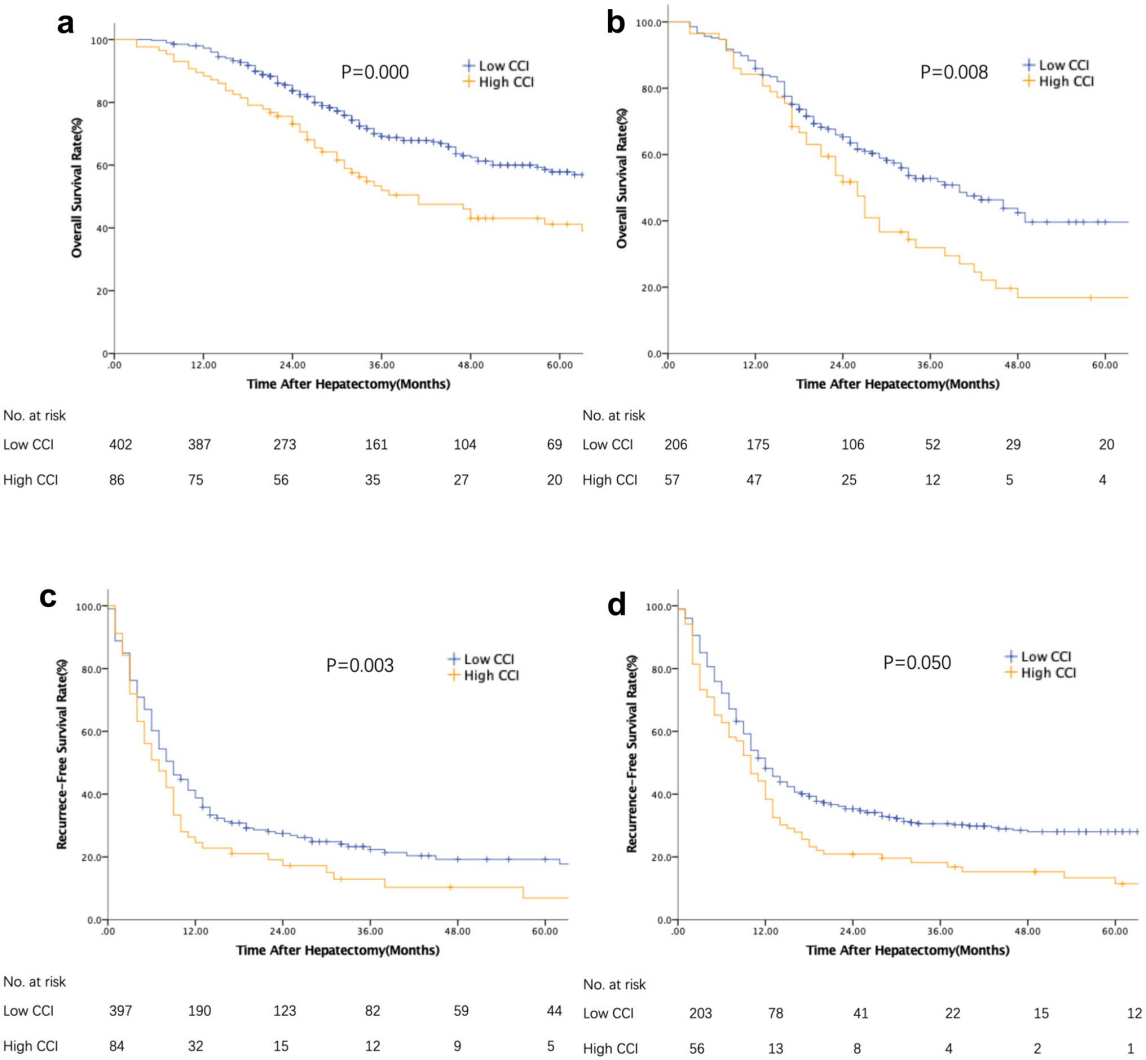
Subgroup analyses of OS and RFS in patients with low and high M-CS according to CCI. **a** OS in patients with low M-CS (*p* = 0.000); **b** RFS in patients with low M-CS (*p* = 0.003); **c** OS in patients with high M-CS (*p* = 0.008); and **d** RFS in patients with high M-CS (*p* = 0.050). OS, overall survival; RFS, recurrence-free survival

Patients were stratified on the basis of the M-CS. Tables 3 and 4 describe the risk factors associated with OS and RFS after liver resection for CRLM in different subgroups. In the subgroup of patients with low M-CS, the median OS and RFS were 40 and 10 months, respectively, for the high CCI group and 109 and 12 months, respectively, for the low CCI group (*P* = 0.000, *P* = 0.003 Fig. 1A, B). Importantly, in the multivariable analysis, OS and RFS were still significantly worse in patients with a high CCI (HR 1.49, 1.03–2.16, *P* = 0.035 and HR 1.32, 1.00–1.75, *P* = 0.048, respectively, Table 3). In the group of patients with high M-CS, although comparisons of the OS and RFS of patients who had high CCI or low CCI favored low CCI (*P* = 0.008, *P* = 0.050 Fig. 1C, D), no significant difference was detected in multivariable analysis (HR 1.44, 0.99–2.10, *P* = 0.054 and HR 1.02, 0.73–1.43, *P* = 0.893, respectively, Table 4).

**Table 3:** Univariable and multivariable analyses for overall survival and recurrence-free survival in patients with low-risk score (M-CS 0–1)

Variables	Overall survival						Recurrence-free survival					
	Univariable			Multivariable			Univariable			Multivariable		
	HR	95% CI	*P*	HR	95% CI	*P*	HR	95% CI	*P*	HR	95% CI	*P*
Sex (female vs. male)	0.87	0.64–1.18	0.356				0.87	0.70–1.08	0.205			
Age (≥ 65 years)	0.91	0.64–1.29	0.591				0.77	0.59–0.99	0.039	0.84	0.64–1.08	0.174
ASA score (III/I,II)	0.95	0.76–1.20	0.682				1.02	0.87–1.20	0.809			
Primary tumor												
Site (right vs. left)	1.38	0.95–2.00	0.091	1.41	0.96–2.06	0.077	1.29	0.98–1.70	0.074	1.33	0.99–1.78	0.059
T category (T3-4 vs. T1-2)	1.50	0.85–2.64	0.161				1.24	0.86–1.79	0.255			
N category (N1-2 vs. N0)	1.33	0.99–1.80	0.061	1.36	1.00–1.85	0.048	1.25	1.01–1.54	0.042	1.22	0.98–1.52	0.071
CRLM characteristics												
Tumor number (multiple)	1.26	0.93–1.72	0.141				1.80	1.43–2.26	0.000	1.30	0.96–1.76	0.085
Maximum tumor size ≥ 5 cm	0.83	0.36–1.89	0.654				1.18	0.68–2.05	0.567			
Distribution (bilobar vs. unilobar)	1.16	0.87–1.56	0.315				1.55	1.25–1.91	0.000	1.06	0.81–1.37	0.683
Ras status (mutation vs. wild type)	0.99	0.63–1.54	0.953				1.10	0.81–1.49	0.531			
Preoperative factors												
Preoperative chemotherapy (yes vs. no)	0.93	0.68–1.28	0.667				0.64	0.51–0.82	0.000	0.78	0.58–1.03	0.083
CEA, ng/mL (< 20 vs. ≥ 20)	1.36	0.97–1.90	0.071	1.53	1.08–2.15	0.016	1.36	1.08–1.72	0.010	1.56	1.22–1.99	0.000
Synchronous metastasis	1.15	0.86–1.55	0.350				1.39	1.13–1.72	0.002	1.07	0.84–1.35	0.592
Extrahepatic metastasis	1.51	1.27–1.79	0.000	1.57	1.32–1.86	0.000	1.24	1.08–1.42	0.002	1.24	1.08–1.42	0.003
Surgical factors												
Intraoperative ablation	0.61	0.35–1.08	0.090	0.72	0.41–1.28	0.264	2.04	1.56–2.66	0.000	1.87	1.39–2.52	0.000
Intraoperative blood transfusion	1.65	1.02–2.65	0.040	1.33	0.78–2.28	0.297	1.21	0.82–1.78	0.327			
Major resection	1.53	1.06–2.22	0.024	1.26	0.85–1.86	0.250	1.22	0,92–1.63	0.173			
Resection margin width, mm (< 1 vs. ≥ 1)	1.40	1.02–1.92	0.039	1.64	1.18–2.26	0.003	1.23	0.98–1.55	0.080	1.28	1.00–1.63	0.047
High CCI	1.78	1.28–2.47	0.001	1.49	1.03–2.16	0.035	1.46	1.13–1.89	0.004	1.32	1.00–1.75	0.048
Infectious complication	1.20	0.98–1.45	0.074	1.05	0.85–1.31	0.650	1.09	0.94–1.26	0.273			
Major complication	0.99	0.78–1.26	0.963	1.46	1.00–2.13	0.049	1.02	0.86–1.22	0.826			
Adjuvant chemotherapy (yes vs. no)	0.78	0.56–1.07	0.121				0.82	0.65–1.04	0.097	0.61	0.45–0.84	0.002

**Table 4:** Univariable and multivariable analyses for overall survival and recurrence-free survival in patients with high-risk score (M-CS 2–3)

Variables	Overall survival						Recurrence-free survival					
	Univariable			Multivariable			Univariable			Multivariable		
	HR	95% CI	*P*	HR	95% CI	*P*	HR	95% CI	*P*	HR	95% CI	*P*
Sex (female vs. male)	0.88	0.63–1.23	0.458				1.01	0.77–1.34	0.926			
Age (≥ 65 years)	1.03	0.70–1.52	0.895				1.15	0.83–1.60	0.388			
ASA score(III/I,II)	0.94	0.74–1.20	0.613				1.10	0.91–1.33	0.349			
Primary tumor												
Site (right vs. left)	0.94	0.64–1.37	0.734				0.91	0.66–1.25	0.565			
T category (T3-4 vs. T1-2)	1.38	0.65–2.95	0.408				1.50	0.82–2.75	0.167			
N category (N1-2 vs. N0)	1.04	0.51–2.12	0.922				1.50	0.79–2.82	0.214			
CRLM characteristics												
Tumor number (multiple)	1.21	0.86–1.71	0.269				1.70	1.27–2.26	0.000	1.36	0.91–2.02	0.132
Maximum tumor size ≥ 5 cm	1.08	0.76–1.53	0.671				1.07	0.80–1.43	0.638			
Distribution (bilobar vs. unilobar)	1.35	0.97–1.87	0.077				1.64	1.25–2.15	0.000	1.25	0.87–1.80	0.232
Ras status (mutation vs. wild type)	1.34	0.91–1.99	0.143				1.23	0.89–1.70	0.209			
Preoperative factors												
Preoperative chemotherapy (yes vs. no)	1.05	0.74–1.49	0.794				0.89	0.66–1.20	0.429			
CEA, ng/mL (< 20 vs. ≥ 20)	1.32	0.95–1.83	0.103				1.58	1.21–2.08	0.001	1.48	1.13–1.96	0.005
Synchronous metastasis	1.18	0.85–1.65	0.320				1.57	1.19–2.06	0.001	1.31	0.96–1.79	0.084
Extrahepatic metastasis	1.25	1.02–1.54	0.034	1.27	1.03–1.57	0.026	1.12	0.94–1.33	0.206			
Surgical factors												
Intraoperative ablation	0.83	0.47–1.46	0.512				1.07	0.71–1.62	0.741			
Intraoperative blood transfusion	1.40	0.85–2.29	0.187				1.11	0.70–1.76	0.665			
Major resection	1.34	0.90–1.97	0.147				1.15	0.82–1.61	0.432			
Resection margin width, mm (< 1 vs. ≥ 1)	1.28	0.90–1.83	0.176				1.11	0.82–1.51	0.487			
High CCI	1.61	1.12–2.30	0.010	1.44	0.99–2.10	0.054	1.35	0.99–1.86	0.060	1.02	0.73–1.43	0.893
Infectious complication	1.23	1.01–1.49	0.036	1.17	0.96–1.43	0.120	1.16	0.98–1.37	0.084	1.02	0.86–1.22	0.805
Major complication	1.16	0.94–1.42	0.179				1.08	0.90–1.30	0.384			
Adjuvant chemotherapy (yes vs. no)	0.73	0.52–1.03	0.076	0.71	0.50–1.01	0.057	0.71	0.53–0.95	0.022	0.65	0.48–0.88	0.005

## Discussion

The present retrospective study analyzed the long-term prognostic implications of postoperative complications among patients with CRLM after accounting for the impact of biologic (RAS status) and tumor burden variables (tumor size and lymph-node metastases). Interestingly, infective complications and major complications were not associated with inferior OS and RFS in the overall cohort or in the subset of patients with different M-CSs. In contrast, a high CCI was strongly associated with decreased OS in the overall cohort. The deleterious effect of the high CCI after hepatectomy varied in different M-CS groups. We found that the CCI might be an independent prognostic factor of patients with low M-CS, whereas the CCI did not affect long-term survival in high M-CS patients. Thus, the CCI may be a suitable definition of postoperative complications to predict oncological outcome in patients with CRLM. Perhaps, more importantly, our analyses indicated that the CCI was not specifically associated with decreased RFS and OS in patients with high M-CS. Patients with high M-CS had shorter OS and RFS, and previous studies support the concept that postoperative complications can drive prognosis after resection of CRLM. Therefore, it typically seems questionable to regard hepatectomy as the treatment of choice in high M-CS patients with a high risk of postoperative complications. Our findings indicate that postoperative complications are not a decisive factor to justify the use of hepatectomy for CRLM in patients with high M-CS.

Postoperative complications is a well-known predictor of prognosis after CRLM resection. The inflammatory cytokines and growth factors induced by complications [16, 17] may be involved in the main mechanisms underlying poor prognosis in patients with complications. However, the most suitable definition of postoperative complications to predict long-term survival outcomes remains unclear. Therefore, our study evaluated the prognostic impact of several popular definitions. Some differences between our study and other reports were noted [6, 8, 9]. We found that infective complications and major complications did not affect the short-term or long-term survival of the patients. High CCI was not associated with RFS but was associated with OS. A possible reason is that CCI uses all complications for the calculation [18, 19]. In contrast, in patients with major complications, only the most severe complication is used [20], and infective complications only take one type of complication into account. Thus, CCI may more accurately reflect the levels of inflammatory cytokines and growth factors that could stimulate the growth of residual cancer cells. Another possible reason is that a high CCI may worsen the general condition, which may lead to poor tolerance for additional therapy.

Previous studies have shown that surgery may promote the formation of new metastases and the growth of preexisting micrometastases [21–23]. Although the mechanism has not yet been completely elucidated, the inflammation induced by postoperative complications and cellular immunity suppression [24, 25] produced by extensive surgery may be responsible for the negative effect of surgery. Therefore, we hypothesized that tumor biology (micrometastasis was informative of the underlying tumor biology) and tumor burden (could affect the extent of surgical resection) may directly influence the oncologic effect of complications. M-CS was chosen to verify our hypothesis given that RAS mutational status is the most evaluated biologic marker in patients with CRLM and tumor size or lymph-node metastases may serve as surrogates of tumor burden. As hypothesized, when examining the cohort while accounting for the potential impact of M-CS, a high CCI was independently associated with both RFS and OS among patients with low M-CS, but a high CCI was not predictive of short- and long-term outcomes among patients with CRLM. Thus, in the current era of advancing personalized therapeutic strategies for patients with CRLM, perioperative care to prevent postoperative complications is very important to improve the survival of patients with low M-CS. Recently, some studies demonstrated that laparoscopic hepatectomy decreased the risk of complications and was associated with a significantly reduced hospital stay compared with open hepatectomy for CRLM [26, 27]. Therefore, laparoscopic hepatectomy may be the preferential surgical procedure for low M-CS patients.

Compared to the Memorial Sloan Kettering Clinical Score, M-CS involves three variables and showed good discriminatory power to identify patients at risk of recurrence and long-term mortality [13]. This score was able to stratify patients into low- and high-risk prognostic groups. All patients in high M-CS presented at least two of the three high-risk factors and these patients were characterized by a certain homogeneity in the tumor-related aspects. Therefore, after risk adjustment by Cox regression, CEA > 20 and lack of adjuvant chemotherapy were the only independent predictors of decreased RFS and extrahepatic metastasis was the only predictor of increased mortality risk for OS.

The present study has several limitations. For example, one hypothesis of the influence of postoperative complications is that it may lead to delayed adjuvant chemotherapy and therefore influence survival [28]. We could not calculate the median time to adjuvant chemotherapy due to a lack of detailed information on postoperative chemotherapy given that many of our patients came to us from other provinces and received postoperative chemotherapy at another institution. Furthermore, this single-center design makes the study susceptible to selection bias. However, a single center can provide a high degree of standardization of patient selection, operative techniques, and postoperative care. In addition, we recognize a major limitation of this study given its retrospective nature. Changes and improvements in the chemotherapy, diagnostic, and surgical techniques may have influenced the survival data, because the patients underwent hepatectomy during the 11-year time span of this study.

In summary, in contrast with most previous studies, it was demonstrated that major or infectious complications were not associated with decreased RFS and OS in the overall cohort. A high CCI was significantly associated with decreased OS in the whole cohort and with an increased risk of tumor recurrence and worse OS in patients with low M-CS. In contrast, patients with high M-CS had similar RFS and OS irrespective of whether they suffered postoperative complications. Our findings demonstrate that tumor biology and tumor burden mitigate the adverse impact of postoperative complications on long-term survival. Surgeons should try to prevent complications from developing a high CCI in patients with low M-CS.

## Supplementary Information

Below is the link to the electronic supplementary material.

Supplementary file1 (DOCX 1193 KB)

## Data Availability

All data used during the study are available from the corresponding author by reasonable request.
