# Supplementary material for: Postoperative complications predict poor outcomes only in patients with a low modified clinical score after resection of colorectal liver metastases: a retrospective cohort study
Source: Updates Surg. 2022 Jul 20;74(5):1601–10. doi: 10.1007/s13304-022-01312-7 (PMC9481509; doi:10.1007/s13304-022-01312-7)
Supplement: Supplementary file 1 — Supplementary file1 (DOCX 1193 KB) [file 13304_2022_1312_MOESM1_ESM.docx]

**SUPPLEMENTARY MATERIAL TO:**

**The Survival Impact Of Postoperative Complications After Colorectal Liver Metastases Resection Varies According To Modified Clinical Score**

**Hong-Wei Wang, MD ^1^, Ke-Min Jin, MD ^1^ ,Juan Li ^1^, Kun Wang, MD ^1^,and Bao-Cai Xing, MD ^1^**

Hepatopancreatobiliary Surgery Department I, Key Laboratory of Carcinogenesis and Translational Research, Ministry of Education, Peking University School of Oncology, Beijing Cancer Hospital and Institute, Haidian District, Beijing, China

**Table of Contents**

**Supplementary table 1.** Calculation of Modified Clinical Score…………………..2

**Supplementary table 2.** Comparison of characteristics between patients had high and low CCI and with and without postoperative infective or major complications….......3

**Supplementary table 3.** Univariable and multivariable analysis for overall survival in whole study cohort…………………………………………………………….…..4-5

**Supplementary table 4.** Univariable and multivariable analysis for recurrence-free survival in whole study cohort………………………………………………….…6-7

**Supplementary figure 1.** Kaplan-Meier curves of overall survival in patients with high CCI , low CCI(a), Inf‐POC，Non-infPOC(b), C-D grade ≥III and C-D grade ≤II (c)after resection of liver metastasis from CRC in the whole cohort. ………….……….…….8

**Supplementary figure 2.** Kaplan-Meier curves of recurrence-free survival in patients with high CCI , low CCI(a), Inf‐POC，Non-infPOC(b), C-D grade ≥III and C-D grade ≤II (c)after resection of liver metastasis from CRC in the whole cohort. ………….….9

Supplementary table 1. Calculation of Clinical Risk Score

| Variables | Score |
| --- | --- |
| Nodal status of primary |  |
| Positive | 1 |
| Negative | 0 |
| Ras syatus |  |
| mutated | 1 |
| wild | 0 |
| Size of the largest tumor |  |
| > 5cm | 1 |
| < 5cm | 0 |

Supplementary table 2. Comparison of characteristics between patients had high and low CCI and with and without postoperative infective or major complications

| Variables | CCI | | | | infective complications | | | Major complications | | |  |
| --- | --- | --- | --- | --- | --- | --- | --- | --- | --- | --- | --- |
|  | High(143) | Low(608) | P | With(127) | | Without(624) | P | With(87) | Without(664) | P | |
| Sex  Male(%) | 82(57.3) | 403(66.3) | 0.052 | 80(63.0) | | 405(64.9) | 0.685 | 50(57.5) | 435(65.5) | 0.153 | |
| Age  median (i.q.r), yr | 58(50-64) | 58(51-64) | 0.412 | 58(51-64) | | 59(51-65) | 0.400 | 55(48-63) | 58(51-64) | 0.097 | |
| Charlson Index scores  median ( i.q.r ) | 7(7-8) | 8(7-8) | 0.460 | 8(7-9) | | 8(7-8) | 0.199 | 7(6-8) | 8(7-8) | 0.125 | |
| ASA，I/II/III  Primary tumor | 74/55/14 | 330/216/62 | 0.752 | 63/48/16 | | 341/223/60 | 0.461 | 43/39/5 | 361/232/71 | 0.117 | |
| Site (right sided%) | 48(33.6) | 91(15.0) | 0.001 | 37(29.1) | | 102(16.3) | 0.002 | 31(35.6) | 108(16.3) | 0.000 | |
| T category (T1/2: T3/4) | 13/130 | 52/556 | 0.869 | 13/114 | | 52/572/ | 0.489 | 9/78 | 56/608 | 0.543 | |
| N category (N1-2:N0) | 104/39 | 427/181 | 0.610 | 90/37 | | 441/183 | 1.000 | 67/20 | 464/200 | 0.210 | |
| CRLM characteristics |  |  |  |  | |  |  |  |  |  | |
| Synchronous : metachronous | 98/45 | 299/309 | 0.000 | 80/47 | | 317/307 | 0.015 | 62/25 | 335/329 | 0.000 | |
| Tumor number (multiple) | 111(77.6) | 366(60.2) | 0.000 | 91(71.6) | | 386(61.9) | 0.026 | 70(80.4) | 407(61.1) | 0.000 | |
| Maximum tumor size ≥ 5cm(%) | 25(17.5) | 71(11.7) | 0.070 | 20(15.7) | | 76(12.2) | 0.307 | 17(19.5) | 79(11.9) | 0.059 | |
| Distribution (bilobar) | 86(60.1) | 281(46.2) | 0.003 | 71(55.9) | | 296(47.4) | 0.098 | 52 (59.8) | 315(47.4) | 0.040 | |
| Ras status (mutation : wild) | 50/93 | 213/395 | 0.535 | 47/80 | | 216/408 | 0.611 | 33/54 | 230/434 | 0.552 | |
| Extrahepatic disease(%) | 26(18.2) | 95(15.6) | 0.450 | 29(22.8) | | 92(14.7) | 0.033 | 16(18.4) | 105(15.8) | 0.536 | |
| Preoperative factors |  |  |  |  | |  |  |  |  |  | |
| Preoperative chemotherapy (%） | 106(74.1) | 424(69.7) | 0.359 | 97(76.4) | | 433(69.4) | 0.135 | 65(74.7) | 465(70.0) | 0.453 | |
| ≤6 cycles(%） | 119(83.2) | 523(86.0) | 0.428 | 108(85.0) | | 534(85.6) | 0.890 | 71(81.6) | 571(86.0) | 0.261 | |
| ≥2 regimens | 20(14.0) | 63(10.4) | 0.235 | 14(11.0) | | 69(11.1) | 1.000 | 15(17.2) | 68(10.2) | 0.067 | |
| Use of Oxaliplatin | 87(60.8） | 305(50.2) | 0.025 | 75(59.1) | | 317(50.8) | 0.098 | 53(60.9) | 339(51.1) | 0.088 | |
| Use of Irinotecan | 42(29.3) | 169(27.6) | 0.756 | 37(29.1) | | 174(27.9) | 0.829 | 25(28.7) | 186(28.0) | 0.899 | |
| Use of anti-EGFR agent | 29(20.3) | 121(19.9) | 0.908 | 22(17.3) | | 128(20.5) | 0.466 | 18(20.7) | 132(19.9) | 0.887 | |
| Use of bevacizumab | 27(18.9) | 111(18.3) | 0.905 | 28(22.0) | | 110(17.6) | 0.258 | 17(19.5) | 121(18.2) | 0.769 | |
| CEA, median ( i.q.r)ng/mL | 11(4.7-38.4) | 7.7(3.3-23.9) | 0.021 | 11.1(4.5-39.1) | | 7.8(3.4-23.0) | 0.016 | 11.1(3.8-38.4) | 8.0(3.5-23.3) | 0.130 | |
| Hepatic resection |  |  |  |  | |  |  |  |  |  | |
| Operative time, median (range),min | 240(185-315) | 175(131-235) | 0.000 | 240(180-313) | | 175(135-240) | 0.000 | 240(180-313) | 180(135-240) | 0.000 | |
| Plus ablation | 16(11.2) | 93(15.3) | 0.236 | 16(12.6) | | 93(14.9) | 0.581 | 12(13.8) | 97(14.6) | 1.000 | |
| Intraoperative transfusion | 40(28.0) | 21(3.5) | 0.000 | 30(23.6) | | 31(4.97) | 0.000 | 21(24.1) | 40(6.0) | 0.000 | |
| Blood loss(range),ml | 200(100-300) | 100(100-200) | 0.000 | 200(100-300) | | 150(100-200) | 0.000 | 200(100-300) | 150(100-200) | 0.000 | |
| R1 Resection | 43(30.1) | 155(25.5) | 0.291 | 39(30.7) | | 159(25.5) | 0.226 | 21(24.1) | 177(26.7) | 0.025 | |
| Major resection | 47(32.9) | 71(11.7) | 0.000 | 25(19.7) | | 93(14.9) | 0.182 | 29(33.3) | 89(13.4) | 0.000 | |
| Adjuvant chemotherapy | 105(73.4) | 440(72.3) | 0.836 | 93(73.2) | | 45272.4) | 0.913 | 63(72.4) | 482(72.6) | 1.000 | |

Table 3. Prognostic Factors for Overall Survival in whole cohort on Univariate and Multivariate Analysis (Cox Proportional Hazard Model)

|  | **Univariate** | | |  | **Multivariate** | | |  |
| --- | --- | --- | --- | --- | --- | --- | --- | --- |
|  | **HR (95% CI)** | ***p*** |  | | **HR (95% CI)** | | ***p*** | |
| Patient age>65 | 0.95 (0.73-1.23) | 0.695 |  | | - | |  | |
| Female gender | 0.84 (0.67-1.06) | 0.844 |  | | - | |  | |
| ASA |  |  |  | |  | |  | |
| I/II | Ref |  |  | |  | |  | |
| III | 0.93(0.79-1.10) | 0.405 |  | |  | |  | |
| Primary tumor location |  |  |  | |  | |  | |
| Left-sided primary | Ref |  |  | | Ref | |  | |
| Right-sided primary | 1.26 (0.96-1.64) | 0.091 |  | | 1.01 (0.77-1.34) | | 0.937 | |
| Primary tumor stage |  |  |  | |  | |  | |
| T1 & T2 | Ref |  |  | |  | |  | |
| T3 &T4 | 1.56 (0.99-2.46) | 0.054 |  | | 1.35 (0.85-2.15) | | 0.202 | |
| Lymph Node metastasis | 1.60 (1.24-2.07) | 0.000 |  | | 1.46 (1.13-1.89) | | 0.004 | |
| Preoperative chemotherapy | 0.97 (0.77-1.23) | 0.809 |  | |  | |  | |
| CEA >20 ng/dl | 1.50 (1.20-1.89) | 0.001 |  | | 1.38 (1.08-1.76) | | 0.010 | |
| Synchronous liver metastases | 1.15 (0.93-1.44) | 0.203 |  | |  | |  | |
| Tumor number (multiple) | 1.22 (0.97-1.54) | 0.086 |  | | | 1.04(0.77-1.41) | 0.774 | |
| Maximum tumor size ≥ 5cm | 1.46 (1.08-1.96) | 0.014 | | | 1.19(0.86-1.64) | | 0.306 | |
| Bilateral liver disease | 1.21 (0.97-1.50) | 0.088 |  | | 1.08(0.82-1.43) | | 0.590 | |
| RAS/BRAF status  Wild-type tumors | Ref |  |  | | Ref | |  | |
| Mutated | 1.65 (1.32-2.07) | 0.000 |  | | 1.70 (1.35-2.13) | | 0.000 | |
| Extrahepatic disease | 1.40 (1.23-1.60) | 0.000 |  | | 1.44(1.26-1.65) | | 0.000 | |
| Red blood cell transfusion | 1.58(1.12-2.22) | 0.009 |  | | 1.14 (0.78-1.67) | | 0.501 | |
| Major resection | 1.52 (1.17-1.99) | 0.002 |  | | 1.14(0.77-1.67) | | 0.515 | |
| Intraoperative ablation | 0.68 (0.45-1.02) | 0.060 |  | | 0.73(0.48-1.12) | | 0.145 | |
| R1 resection | 1.34 (1.06-1.70) | 0.014 |  | | 1.40 (1.09-1.78) | | 0.007 | |
| High CCI(≥26.2) | 1.76 (1.38-2.25) | 0.000 |  | | 1.51(1.14-2.00) | | 0.004 | |
| Infectious complication | 1.49 (1.15-1.94) | 0.003 |  | | 1.25(0.95-1.65) | | 0.119 | |
| Major complication | 1.12 (0.96-1.31) | 0.163 |  | |  | |  | |
| Adjuvant chemotherapy | 0.75 (0.59-0.95) | 0.016 |  | | 0.75 (0.59-0.96) | | 0.020 | |

Table 4. Prognostic Factors for recurrence-free survival in whole cohort on Univariate and Multivariate Analysis (Cox Proportional Hazard Model)

|  | **Univariate** | | |  | **Multivariate** | | | |  |  |
| --- | --- | --- | --- | --- | --- | --- | --- | --- | --- | --- |
|  | **HR (95% CI)** | ***p*** |  | | **HR (95% CI)** | | | ***p*** | | |
| Patient age>65 | 0.876 (0.71-1.06) | 0.178 |  | | - | | |  | | |
| Female gender | 0.91 (0.77-1.08) | 0.270 |  | | - | | |  | | |
| ASA |  |  |  | |  | | |  | | |
| I/II | Ref |  |  | |  | | |  | | |
| III | 1.03(0.92-1.17) | 0.594 |  | |  | | |  | | |
| Primary tumor location |  |  |  | |  | | |  | | |
| Left-sided primary | Ref |  |  | |  | | |  | | |
| Right-sided primary | 1.15 (0.93-1.41) | 0.197 |  | |  | | |  | | |
| Primary tumor stage |  |  |  | |  | | |  | | |
| T1 & T2 | Ref |  |  | |  | | |  | | |
| T3 &T4 | 1.35 (0.99-1.85) | 0.061 |  | | 1.15 (0.84-1.59) | | | 0.389 | | |
| Lymph Node metastasis | 1.37 (1.14-1.65) | 0.001 |  | | 1.30 (1.07-1.57) | | | 0.007 | | |
| Preoperative chemotherapy | 0.72 (0.60-0.87) | 0.001 |  | | 0.92 (0.74-1.14) | | | 0.419 | | |
| CEA >20 ng/dl | 1.51 (1.27-1.80) | 0.000 |  | | 1.48 (1.23-1.79) | | | 0.000 | | |
| Synchronous liver metastases | 1.45 (1.23-1.72) | 0.000 |  | | 1.24 (1.03-1.49) | | | 0.024 | | |
| Tumor number (multiple) | 1.73 (1.45-2.07) | 0.000 |  | | | 1.34 (1.05-1.71) | 0.018 | | |  |
| Maximum tumor size ≥ 5cm | 1.26 (1.00-1.60) | 0.053 | | | 1.19(0.92-1.53) | | | 0.182 | | |
| Bilateral liver disease | 1.55 (1.31-1.83) | 0.000 |  | | 1.15(0.93-1.41) | | | 0.194 | | |
| RAS/BRAF status  Wild-type tumors | Ref |  |  | | Ref | | |  | | |
| Mutated | 1.30 (1.10-1.55) | 0.002 |  | | 1.28 (1.07-1.52) | | | 0.006 | | |
| Extrahepatic disease | 1.20 (1.08-1.33) | 0.001 |  | | 1.22(1.10-1.36) | | | 0.000 | | |
| Red blood cell transfusion | 1.19(0.88-1.60) | 0.253 |  | |  | | |  | | |
| Major resection | 1.22(0.98-1.51) | 0.079 |  | | 1.09(0.87-1.38) | | | 0.449 | | |
| Intraoperative ablation | 1.62 (1.29-2.02) | 0.000 |  | | 1.44(1.13-1.83) | | | 0.003 | | |
| R1 resection | 1.19(0.99-1.43) | 0.066 |  | | 1.11 (0.92-1.34) | | | 0.296 | | |
| High CCI(≥26.2) | 1.44 (1.18-1.76) | 0.000 |  | | 1.16(0.94-1.44) | | | 0.166 | | |
| Infectious complication | 1.35 (1.09-1.66) | 0.006 |  | | 1.14(0.91-1.41) | | | 0.250 | | |
| Major complication | 1.07 (0.94-1.21) | 0.305 |  | |  | | |  | | |
| Adjuvant chemotherapy | 0.78 (0.65-0.93) | 0.006 |  | | 0.69 (0.57-0.84) | | | 0.000 | | |


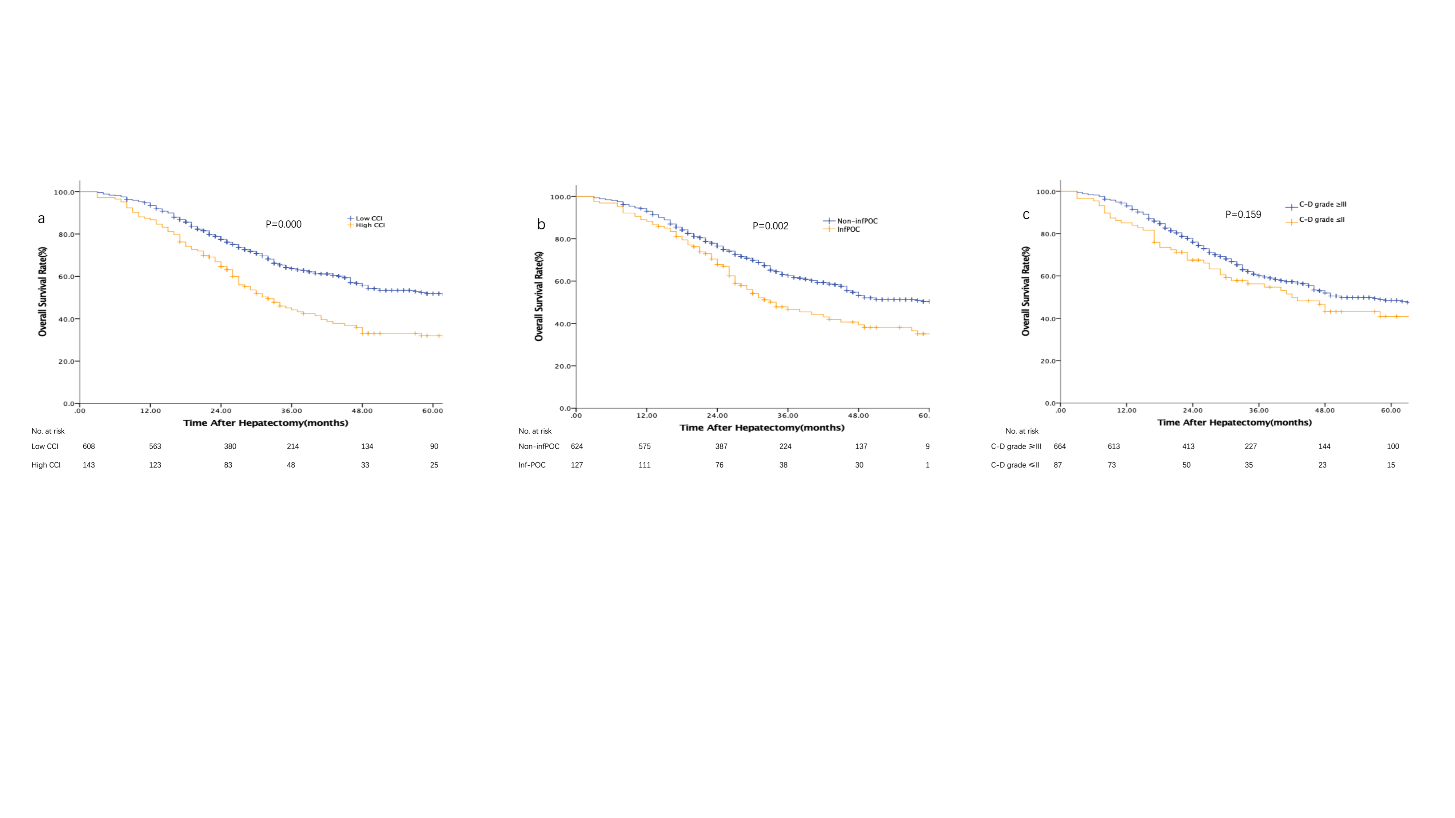


**Supplementary figure 1.** Kaplan-Meier curves of overall survival in patients with high CCI , low CCI(a), Inf‐POC，Non-infPOC(b), C-D grade ≥III and C-D grade ≤II (c)after resection of liver metastasis from CRC in the whole cohort


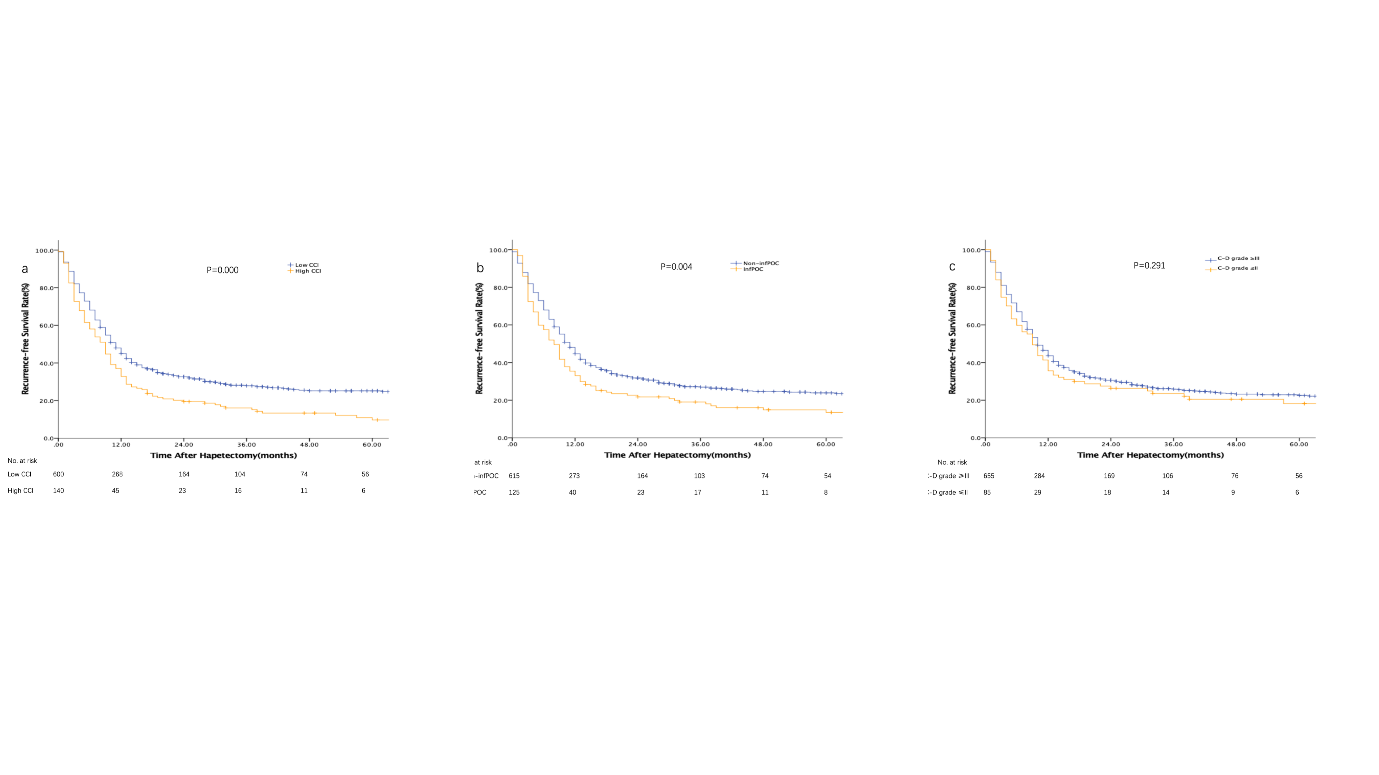


**Supplementary figure 2.** Kaplan-Meier curves of recurrence-free survival in patients with high CCI , low CCI(a), Inf‐POC，Non-infPOC(b), C-D grade ≥III and C-D grade ≤II (c)after resection of liver metastasis from CRC in the whole cohort.

w
